# Effect of growth at low pH on the cell surface properties of a typical strain of *Lactobacillus casei* group

**Published:** 2010-09

**Authors:** M Hossein Nezhad, DJ Stenzel, ML Britz

**Affiliations:** 1 Khorasan Research Institute for Food Science and Technology, Mashad, Iran; 2 Faculty of Science and Technology, Queensland University of Technology, Queensland 4001, Australia; 3 Faculty of Science, Engineering and Technology, University of Tasmania, TAS 7001, Australia

**Keywords:** *Lactobacillus casei*, transmission electron microscopy, cell surface, acidic condition, SDS-PAGE

## Abstract

**Background and Objectives:**

Although members of the *Lactobacillus casei* group are known to survive under acidic conditions, the underlying mechanisms of growth at acidic condition and the impact of low pH on the relative level of protein expression at the cell surface remain poorly studied.

**Material and Methods:**

After confirming the taxonomy of *L. casei* strain GCRL 12 which was originally isolated from cheese and confirmed by 16S rRNA sequence analysis, the impact of acidic pH on growth rate was determined.

**Results:**

Late log-phase cells cultured at pH 4.0 showed obvious changes in Gram staining properties while transmission electron microscopy analysis revealed evidence of structural distortions of the cell surface relative to the controls cultured at pH 6.5. When comparing cytosolic or whole cell preparations on SDS-PAGE, few changes in protein profiles were observed under the two growth conditions. However, analysis of surface protein extracted by 5M LiCl demonstrated changes in the proportions of proteins present in the molecular weight range of 10 to 80 kDa, with some proteins more dominant at pH 6.5 and other at pH 4.

**Conclusion:**

These data suggest that surface proteins of this strain are associated with growth and survival at low pH. The function of these proteins is subject to further investigation.

## INTRODUCTION

*Lactobacillus casei* is amongst the most common isolates of nonstarter lactic acid bacteria (NSLAB) which has applications as acid-producing cultures for milk fermentation and in acceleration or intensification of flavor in certain bacterial ripened cheese varieties. These bacteria have been detected in the human gastrointestinal tract by molecular approaches and have the potential to function as probiotics with several health benefits. The applications imply that *L. casei* are exposed to various environmental sub-optimal conditions. In nature, the ability to respond quickly to stress is essential for these bacteria to survive, but in food manufacturing, for example, during starter handling and storage, bacterial resistance to adverse conditions often provides practical advantages to the food manufacturer.

During Cheddar cheese maturation, the nutritional environment available to sustain growth and viability of the microflora varies considerably, thus NSLAB grow under sub-optimal growth conditions, including low temperature and a pH below that required for maximum growth (1). Like other LAB, when faced with an acidic environment, *L. casei* appear to have evolved some approaches that permit them to survive and grow in such adverse conditions. Although the molecular analysis of lactic acid bacteria in response to pH downshifts are currently largely specified, the majority of interactions between *L. casei* and the environment/s when the growth starts at low pH are uncharacterized. The general aim of this research was to investigate the characterization and morphological changes of one typical strain of *L. casei* occurring when the bacterial inoculums is cultured at low pH with emphasis on the cell surface, together with assessing the protein profile of these bacteria during growth in an acidic environment with constant pH.

## MATERIALS AND METHODS

**Bacterial strains and growth conditions.** The bacteria *L. casei* strain GCRL 46, a typical strain of the *L. casei* group was isolated from cheddar cheese, originally obtained from CSIRO. Bacteria were routinely cultured using MRS (Oxoid, West Heidelberg, Australia) broth or plates at 37°C, under anaerobic conditions (Oxoid jars with Gas Generating Kit BR038B). Stock cultures were transferred into glycerol broths (50% glycerol in MRS) and stored in cryo-vials under oxygen-free nitrogen at -80°C.

**DNA extraction, PCR and partial 16S rRNA gene sequencing.** Chromosomal DNA from *L. casei* strain 46 was isolated according to the instructions of the “UltraClean Microbial DNA Isolation Kit” (MoBio). The genomic DNA was amplified using the primer combination 27F (5′-AGAGTTTGATCCTGGCTCAG-3′) and 519R (5′-GWATTACCGCGGCKGCTG-3′) targeting the 16S rRNA gene (2). A long template PCR kit (Cat# 28104, QIAGEN) was used and amplification of genomic DNA was performed in the automated thermal cycler (PTC-200, Perkin-Elmer-Cetus) using the protocol described by the manufacturer. The PCR condition of the primer pair was as follows: initial denaturation at 95°C for 10 min, followed by 30 cycles of denaturation at 94°C for 10 s, annealing for 30 sec at 55°C, extension at 68°C for 30 sec, followed by a final extension at 68°C for seven min. Preparation of templates was performed using the Beckman Coulter CEQ 2000 Dye terminator sequencing protocol according to manufacturer's instructions and automated sequencing was done at The University of Melbourne, Gilbert Chandler Research Laboratories (GCRL).

**Experimental design and determination of growth.** To determine the initial pH values that limited growth, starter cultures of *L. casei* strain 46 were prepared by inoculating directly from glycerol storage broth into 20 mL of MRS broth and incubating anaerobically (37°C) for 16 h. Aliquots of starter culture were transferred into 200 mL of buffered MRS to give an initial OD_600_ of ∼ 0.15. Buffered MRS was prepared by aseptically mixing the sterile double strength MRS broth and sterile 0.4 M sodium citrate phosphate buffers with the desired pH values. The headspace of cultures was sparged with sterile oxygen-free nitrogen (in-line 0.22 µm filter) for 5 min, the bottles sealed and incubated at 37°C in anaerobic jars. Samples were removed at one hour intervals under nitrogen gas flow and the headspace replaced before resealing the bottles. OD_600_ was recorded hourly to determine the specific growth rate of the cultures at various pH values (3, 4). The maximum growth rate values (*µ*max) were calculated from three independent experiments.

**Acidic growth conditions.** Following initial culture in MRS broth overnight, *L. casei* strain 46 was sub-cultured into 0.2 M Na-citrate phosphate buffered MRS initially adjusted to pH 4.0 for acidic conditions, and incubated at 37°C anaerobically. A control culture was also prepared at pH 6.5. Cultures from late exponential phase were harvested by centrifugation at 5,000 *g*, 4°C, before the pH of control dropped to less than 6.0 (OD_600_ 1).

**Morphological tests using Transmission electron microscopy.** Samples were prepared for transmission electron microscopy (TEM) by standard procedures (5). Briefly, bacterial cells from acidic or control pH were fixed with 3% glutaraldehyde (ProSciTech) fixative (in 0.2 M Na-citrate phosphate buffer pH 6.5). After fixation for one hour at room temperature, cells were washed with buffer, then postfixed in osmium tetroxide, followed by uranyl acetate. The cells were dehydrated in increasing concentrations of ethanol (50, 70, and 90%) and acetone (90 and 100%) and subsequently embedded in Spurr's epoxy resin. Ultrathin sections (50 to 100 nm in thickness) were prepared and collected onto 200-mesh copper grids, contrasted with 1% uranyl acetate and Reynolds lead citrate before being examined and photographed using a JEOL 1200EX transmission electron microscope, operating at 80 kV. Sections were photographed onto Kodak 4489 electron image film, which was processed according to the manufacturer's instructions.

**Sample preparation for protein analysis.** Cells grown under acidic or control conditions were collected at the late exponential phase by centrifugation at 8,000 *g* for 15 min at 4°C after determining the OD_600_ and pH, then washed twice with 40 mM Tris-HCl buffer pH 7.0 followed by sub-cellular fractionation or extraction with LiCl.

**Cell fractionation procedure for whole cell extracts and cytosolic proteins.** Cells from acidic or control pH were suspended in 40 mM Tris-HCl buffer, pH 7.0 and placed in 2 mL capacity screw-top plastic tubes with 0.5 g of 0.1 mm glass beads. Cell lysis was performed by bead beating in a Mini Bead Beater-8 (Biospecs Products Inc) six bursts of 1 min at maximum speed with 2-min intervals on ice. The tubes were brief centrifuged (5000 *g*, 15 min, 4°C) to settle the beads and unbroken cells. The cytosolic fraction was obtained by high speed centrifugation at 22,000 *g* for 30 min, 4°C.

**Extraction of cell surface associated proteins with LiCl.** Cell surface associated proteins from *L. casei* strain 46 were isolated using LiCl based on a method developed by Lortal *et al.* 1992, (6). Cells from late exponential phase were washed twice with deionized water and resuspended in 0.1–0.15 w/v 5 M LiCl, then incubated with gentle shaking at 4 °C for one hour. Bacterial cell suspensions were then pelleted at 22,000 g for 30 min. Soluble LiCl extracts were filtered through 0.2 µm pore size nitrocellulose membranes, then dialyzed against deionized water (4°C) for at least 24 h, to remove any LiCl from the liquid. The cell surface associated proteins were then concentrated for 100 times by laying the dialysis tubes on spectra/gelTM absorbent (Spectrum Laboratories, Inc., rancho Dominguez, CA, USA).

The protein concentration in each sample was determined using the Bio-Rad protein assay kit (Bio-Rad Laboratories Ltd,. Hemel Hempstead, United Kingdom).

**SDS-PAGE analysis.** Proteins were separated based on molecular weight by 12% sodium dodecyl sulphate-polyacrylamide gel electrophoresis (SDS-PAGE) according to the method of Laemmli 1970, (7). Electrophoresis was performed using a vertical slab system (SBS Scientific, USA) at a constant voltage until the bromophenol blue dye reached the bottom of the gel sandwich. After electrophoresis, the protein bands were visualized by Coomassie brilliant blue. The amount protein loaded onto the gels was 20 µg.

## RESULTS

**Identification and characterization of *L. casei* strains.** Partial 16S rRNA gene sequencing was used to confirm the identity of strain 46. Submitting the sequences obtained from the primer combination 27F and 519R to ANGIS using Blast similarity search tool confirmed the bacterial isolate used in this study showed 99% homology to the *L. casei* species included in the Gene bank database.

**Fig. 1:**
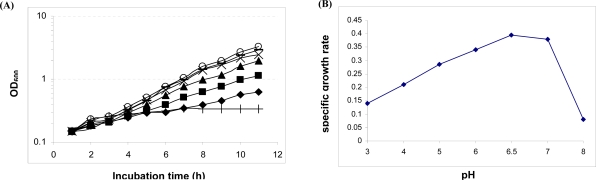
Typical growth curves of *L. casei* strain 46 in 0.2 M Na-citrate phosphate buffered MRS adjusted to initial values of pH 3 (♦), pH 4 (■), pH 5 (▲), pH 6 (×), pH 6.5 (−), pH 7 (O) and pH 8 (+). B: Maximum specific growth rates (µmax) were plotted against the pH of culture medium.

**Growth characterization of *L. casei* strain 46 under acidic conditions.** The impact of pH on the extent of growth was measured from optical density readings (3, 4). The optimum pH of growth was determined by calculating *µ*max values from triplicate measurements of OD_600_ at hourly intervals and used to determine acidic and optimal growth responses (Fig. 1).

The initial pH of the culture had an impact on the specific growth rates. From Fig. 1, the pH 6.5 culture achieved the highest extent of growth, while the overall growth rate declined by either elevating or decreasing the pH of the culture medium. The reduced rates of growth were noted at pH below or above 6.5. The maximum OD_600_ at the end of the incubation time was more than 3.0 at pH 6.5 (viable count 4.3×108 CFU/ml), while it was less than one at pH 4.0 (viable count 1.3×108 CFU/ml), demonstrating that growth was slowed and reduced at the lower pH. OD measurement is an acceptable surrogate for microbial biomass (4) but the relationship between viable count and OD can be influenced by cell characteristics (filament formation, cell shape change due to growth conditions or other changed that may influence the optical properties of cells). It was also noticed that growth was too sparse at pH 3.0 for reliable microbiological and proteomic analysis. Thus, from these results, the best pH for growth of this strain was at 6.5 and pH 4.0. This represented an environment where cells were still able to grow but at a highly reduced rate. The arithmetic growth curves obtained for low-pH cultures are typical of those resulting from partial inhibition by a stress factor (8).

**Fig. 2:**
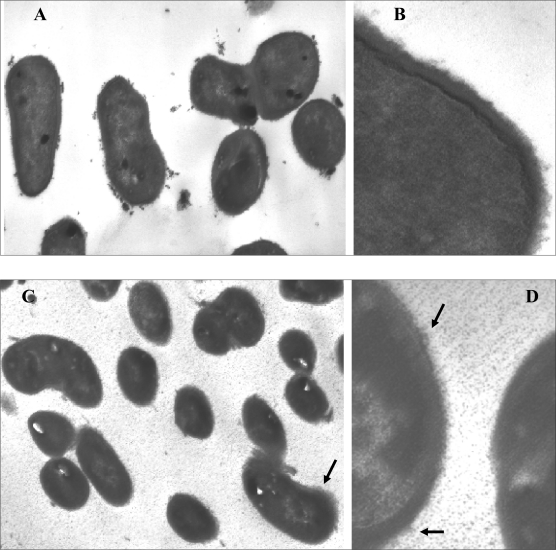
Transmission electron micrographs of bacterial cells from an overnight culture grown in MRS broth buffered at pH 6.5 (A and B) or acidic pH 4.0 (C and D). Lower magnification images (original magnification x25000) show larger cells in culture at pH 6.5 (A), compared to pH 4.0 (C). Higher magnification images (original magnification ×100,000) show details of the cell wall structure, with a more electron dense interface with the environment seen on the cells grown at pH 4.0 (indicated by arrows). Scale bar indicates 200 nm in A and C; 50 nm in B and D.

**Morphological changes of *L. casei* strain 46 at low pH.** TEM analysis revealed some morphological differences between the cells at two growth conditions (Fig. 2) in that the cells grown at acidic pH (4.0) showed some granular material at the cell envelope-environment interface. TEM examination of cells grown at pH 6.5 showed a typical bacterial surface structure, i.e., a clearly defined cell wall and cell membrane (Fig. 2 A, B) with a more electron-dense innermost layer. In contrast, cells under acid stress displayed some alterations in the cell surface structure (Fig. 2 C, D). There was a graded increase in electron density from the inside to the outside with a distinctly different cell wall interface with the environment compared to optimal conditions.

**Fig. 3:**
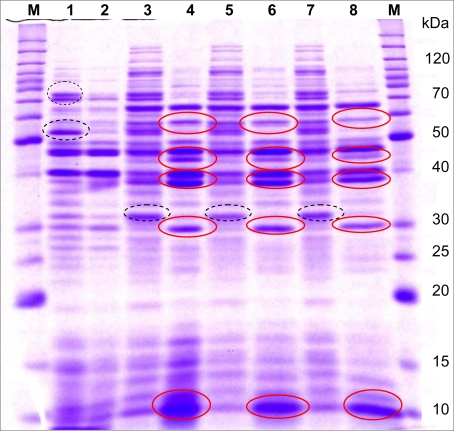
SDS-PAGE profile of surface-associated proteins obtained by three completely different sets of experiments from *Lb. casei* strain 46 compared to cytosolic fraction. Full circles, the bands up-regulated and dashed circles, the bands down-regulated under the acidic pH compared to the optimal growth condition. Lanes: M, protein ladder marker; 1 and 2, cytosolic fraction of growth at pH 6.5 or 4.0 respectively; 3, 5 and 7, 5 M LiCl cell extracts from growth at pH 6.5; 4, 6 and 8, 5 M LiCl cell extracts from growth at pH 4.0.

**Change in 1-D SDS-PAGE profiles of sytosolic and surface-associated proteins at low pH growth conditions.** The protein profiles of *L. casei* strain 46 grown in low or optimal pH were analyzed by SDS-PAGE for supernatant fluids and LiCl cell extracts (Fig. 3). The protein banding patterns observed for cytosolic fractions showed some differences in the relative level of expressions. Over-expression of a number of proteins with estimated molecular weights of approximately 29, 38, 47 and 64 kDa during growth at low pH suggests that the up regulated proteins were related to the cell response during growth under acidic pH. These differences were much more obvious in the protein profile obtained for LiCl cell extracts compared to the cytosolic fraction. In general, the protein profile of LiCl extracts showed that 5 M LiCl removed proteins other than those of the surface layer, presumably those attached to the cell wall. It was obvious that the LiCl extracts contained proteins of similar MW to proteins in the cytosolic fraction.

Although each growth condition was tested in duplicate within each experiment and each set of experimental conditions was tested at least twice, analysis was made to examine the reproducibility of the extraction method of surface-associated proteins using 5 M LiCl treatment. Reproducibility of the extraction methods was high, which was indicated by almost similar 1-D SDS-PAGE protein profile of samples individually prepared, including up-regulation of three bands with molecular weights of 38, 47 and 57 kDa and down-regulation of a band of 34 kDa (Fig. 3).

Although each growth condition was tested in duplicate within each experiment and each set of experimental conditions was tested at least twice, analysis was made to examine the reproducibility of the extraction method of surface-associated proteins using 5 M LiCl treatment. Reproducibility of the extraction methods was high, which was indicated by almost similar 1-D SDS-PAGE protein profile of samples individually prepared, including up-regulation of three bands with molecular weights of 38, 47 and 57 kDa and down-regulation of a band of 34 kDa (Fig. 3).

## DISCUSSION

Although members of the *L. casei* group are known to grow and survive under acidic conditions, the underlying mechanisms remain poorly studies. In this work the cell surface properties of *L. casei* strain 46 was studied to provide an insight into molecular responses of the tested strain during growth at acidic condition. This strain had been isolated from Cheddar cheese as a member of the *L. casei* group and further identified by sequencing. Partial 16S rRNA gene sequencing which was used to identify this strain is considered as the fastest and most unambiguous way to identify lactobacilli, and bacteria in general (9, 10). The primers 27F and 519R utilized in this study have been also used for 16S rRNA gene amplification and sequencing for many years, and they have been proven to be useful in species differentiation in lactobacilli strains (2). However, *L. casei* and *L. paracasei* and *L. zeae* form a closely related taxonomic group within the lactobacilli and identification based on 16S rRNA gene is not able to reveal significant differences between these recently diverged species*;* so, in this case, 16S-rRNA gene sequence analysis can indicate only that strain 46 belongs to this group.

The effect of growth phase on cellular responses has been reported for many bacteria including lactobacilli after environmental stresses (11). The trials conducted and all of analyses were principally performed on cells from late-exponential growth stage to avoid other influences such as adaptation to accumulation metabolites, acidification of the medium by accumulation of lactic acid or induction of a stress response due to starvation. Previous studies mostly used the rich medium MRS while the pH of such a medium is altered during growth of lactic acid bacteria due to acid accumulation. In this work Na-citrate phosphate buffer at a concentration of 0.2 M was chosen to adjust the pH of MRS broth on the basis of the results obtained from optimizing conditions (results are not shown). Buffer concentrations of 0.2 M or higher were found to maintain pH of the culture medium at greater than pH 6.0 during exponential growth phase; however, growth rate was reduced considerably by increasing in the buffer concentration above 0.2 M. This was probably because the change in osmotic conditions exerts a severe osmotic stress on the bacterial cells. Literature presenting the effects of osmotic stress on bacterial growth behavior show similar effects (12, 13)

Although the growth was limited, *L. casei* adapted in acidic pH while a consistent set of proteins was upor down-regulated at low pH. Analysis of these protein samples allowed detection of differentially expressed bands that were related to either cytosolic fractions or were found in cell surface proteins. Single step procedure with 5 M LiCl could be used exclusively to extract surface proteins, as this appeared to give the best chance of removing all proteins exterior to the cell wall, and thus give a more complete picture of this subset of the proteome. A diverse body of literature supports this methodological decision for the removal of surface proteins using 5 M LiCl (14–16). Upregulated protein bands of approximately 29, 38, 47, 57 and 64 kDa in 5 M LiCl were extracted from culture of low pH which either were not expressed, or were present in a lower quantity, in the cytosolic fraction. These were assumed to be surface-associated proteins, according to the methodology. A similar assumption was derived for the down-regulated band of ∼34 kDa. However, it has to be considered that while the surface located proteins are the most abundant molecules in the LiCl extracts, many other proteins are present as minor ones. Some bands presented at higher densities in 5 M LiCl extracts may represent cell wall binding proteins intimately attached to the underlying peptigoglycan. However, a number of proteins extracted by this method are expected to arise from the cytoplasm as a result of cell lysis during the extraction method. The LiCl extracts were concentrated about 100-fold, so that even small levels of cell lysis would result in high representation of cytosolic proteins in those extracts.

Transmission electron microscopy observations also provided evidence of structural distortions under acidic condition. The alterations at low pH were mostly related to the cell surface compared to a clearly defined cell wall and cell membrane (Fig. 2 A and B) which has been observed in other lactobacilli grown under normal growth condition (17). Changes occurred to the cell surface indicating that the cell membrane plays a role in adaptation of *L. casei* strain 46 when it was grown at acidic condition. The cell envelope of bacteria is a shield against subenvironmental conditions and there is growing evidence showing that changes in membranes occur during stress responses. Morphological changes (revealed by EM analysis) have been reported for *L. acidophilus* under freeze-thawing stress (18) and some other lactic acid bacteria in response to bile acid (11); therefore, growth at acidic environment with initial low pH may have morphologically similar impact on the cell surface compared to sharp environmental changes. It also has been documented that *L. casei* shifts the fatty acid composition of the membrane in response to low pH (19). Up-regulation of genes involved in cell wall biogenesis and lipid metabolism in *L. reuteri* have confirmed that cell envelope alterations are important for the stress response in this bacterium, and similar responses are likely to occur in other closely related bacterial species (20).

In conclusion, analysis of microscopic changes and examination of the cell morphology following growth at low pH together with the initial proteomic observations at the cell surface area indicate that the cell surface alterations are important for adapting to conditions that are normally faced during manufacturing and environmental conditions. The tested strain of *L. casei* group appeared to change the composition of the cell surface in order to cope with the growth environment. Further analysis by 2-DE and MALDI-TOF-TOF would be needed for a better separation of proteins and more accurate estimation of sizes, which are being undergoing. Biochemical characterization and detailed studies of the molecular mechanism and cellular roles of these proteins are required to elucidate their role in bacterial response to acidic environments.
